# Artificial Intelligence Paradigms for Next-Generation Metal–Organic Framework Research

**DOI:** 10.1021/jacs.5c08214

**Published:** 2025-06-24

**Authors:** Aydin Ozcan, François-Xavier Coudert, Sven M. J. Rogge, Greta Heydenrych, Dong Fan, Antonios P. Sarikas, Seda Keskin, Guillaume Maurin, George E. Froudakis, Stefan Wuttke, Ilknur Erucar

**Affiliations:** 1 TUBİTAK Marmara Research Center, Materials Technologies, Gebze, Kocaeli 41470, Türkiye; 2 Gebze Technical University, Kocaeli Gebze 41400, Türkiye; 3 Chimie ParisTech, PSL University, CNRS, Institut de Recherche de Chimie Paris, Paris 75005, France; 4 Center for Molecular Modeling (CMM), 26656 Ghent University, Technologiepark-Zwijnaarde 46, Ghent 9052, Belgium; 5 Research Commons Building 4501, Suite 190, Research Triangle Park, North Carolina 27709, United States; 6 ICGM, Univ. Montpellier, CNRS, ENSCM, Montpellier F-34293, France; 7 Department of Chemistry, 37777 University of Crete, Voutes Campus, Heraklion, Crete 70013, Greece; 8 Department of Chemical and Biological Engineering, 52979 Koç University, Rumelifeneri Yolu, Sariyer, Istanbul 34450, Türkiye; 9 Academic Centre for Materials and Nanotechnology, 49811 AGH University of Krakow, Krakow 30-059, Poland; 10 Department of Chemistry, United Arab Emirates University, Al-Ain 15551, United Arab Emirates; 11 Faculty of Engineering, 155531 Ozyegin University, Cekmekoy, Istanbul 34794, Türkiye

## Abstract

After the development of the famous “Transformer” network architecture and the meteoric rise of artificial intelligence (AI)-powered chatbots, large language models (LLMs) have become an indispensable part of our daily activities. In this rapidly evolving era, “all we need is attention” as Google’s famous transformer paper’s title [Vaswani et al., *Adv. Neural Inf. Process. Syst.* **2017**, 30] implies: We need to focus on and give “attention” to what we have at hand, then consider what we can do further. What can LLMs offer for immediate short-term adaptation? Currently, the most common applications in metal–organic framework (MOF) research include automating literature reviews and data extraction to accelerate the material discovery process. In this perspective, we discuss the latest developments in machine-learning and deep-learning research on MOF materials and reflect on how their utilization has evolved within the LLM domain from this standpoint. We finally explore future benefits to accelerate and automate materials development research.

## Graphical abstract

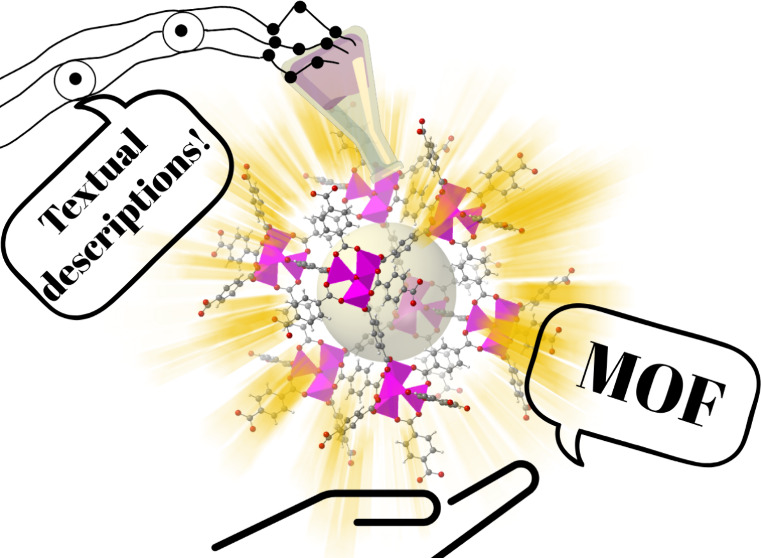

## Introduction

The combinatorial nature of metal–organic frameworks (MOFs) results in a vast chemical toolset and gigantic materials space, offering researchers a theoretically infinite number of candidate materials to choose from for applications spanning from gas storage and separation,^1,2^ to drug delivery.^3–5^ Given the diversity of this enormous chemical space, it is important to reflect on how we can explore this space efficiently in search of the “top” material for a given application. Data-driven techniques (represented in Figure 1) have emerged in recent years as the primary tool for streamlining the identification of the top MOFs. As shown in Figure 1, machine learning (ML) and deep learning (DL) studies are diverse. On the other hand, large language model (LLM) applications of MOFs are still limited^6–27^ but have been increasing rapidly in the last two years.

**Figure 1:**
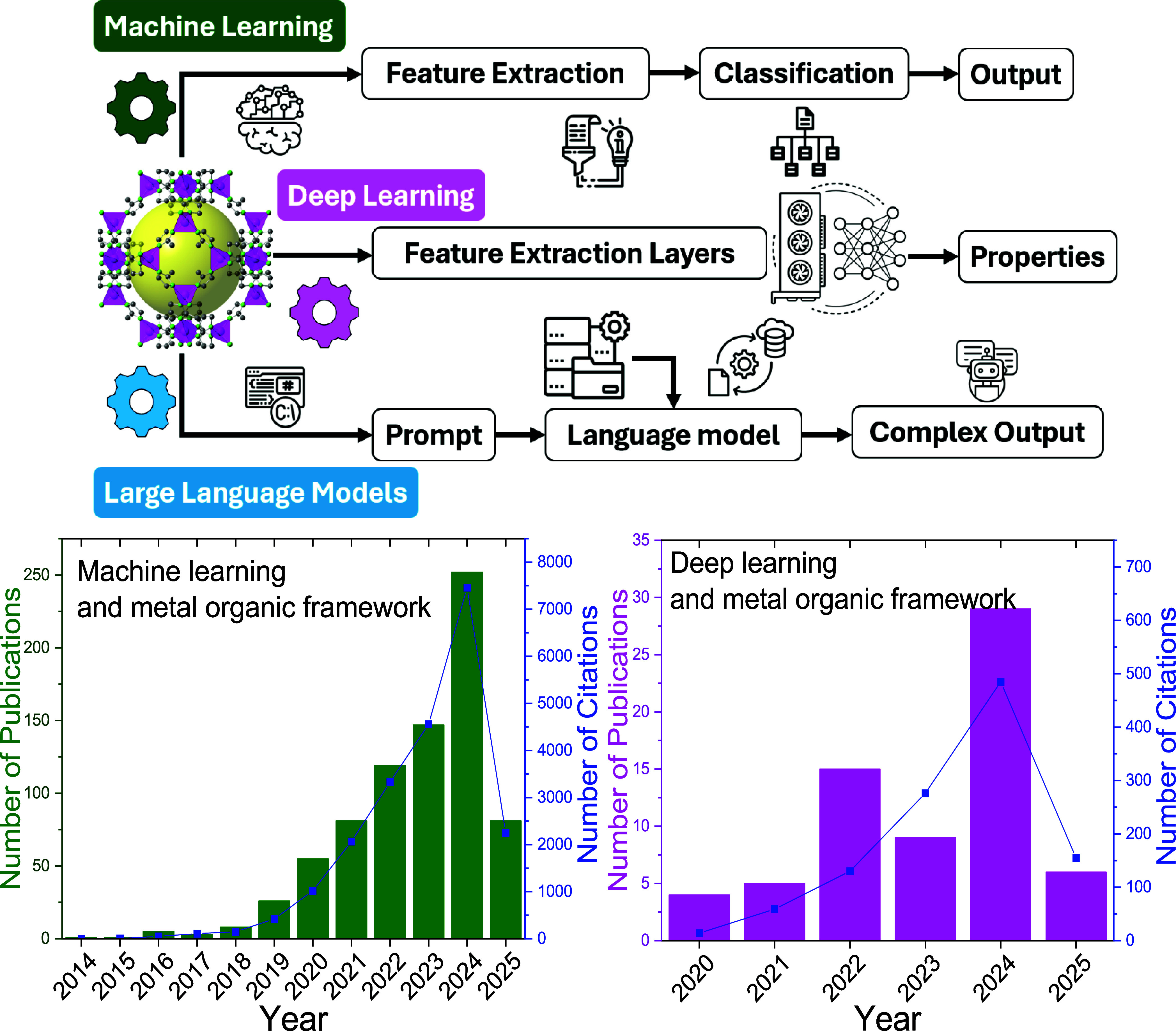
Schematic representation of data-driven methods and their usage in MOF research. Number of publications and their citations featuring the terms “machine learning” and metal organic framework or “deep learning” and metal organic framework in their topics. Accessed: 2025–04–03 from Web of Science.

Additional to the data-centric *“tour de force”* of ML methodologies, the applicability of ML tools in the field of MOF research also appears in the development of machine learning potentials (MLPs), which provide a novel approach to accurately capturing complex interactions with near quantum mechanical precision, while dramatically reducing computational costs for acquiring high-quality data sets, a key ingredient to further train reliable ML-predictive models.^28–39^

In this perspective, we begin our journey by exploring the use of ML methods for predicting structure–property relationships in MOFs. We then discuss the transformative role of DL and LLMs in MOF research, emphasizing their potential to revolutionize the design of novel MOFs with tailored properties on demand. The capability of MLPs to deliver highly accurate predictions, obtained from molecular simulations of MOFs under diverse conditions, is also highlighted. Ensuring easy access to data from diverse material databases, models, and user-friendly tools is crucial for facilitating the widespread adoption of data-driven methods in MOF research and broadening their impact beyond specialized experts to the wider material research community.

## Insights from Early AI-Driven Structure–Property Predictions for MOFs

We are now in an era where data science meets computer simulations. Figure 2 shows the interplay within the overall AI paradigm for the progress of MOF research. High-throughput computational screening (HTCS) approaches based on molecular simulations of MOFs have been important in evaluating large numbers of MOFs (126,800 experimental MOF structures are deposited in the Cambridge Structural Database (CSD),^40^ and trillions of hypothetical MOF structures have been created). In addition, these methods provide molecular-level insights into materials’ properties by complementing and directing the experimental studies.^41^ However, HTCS has become too slow and expensive to explore this materials space effectively and efficiently.

**Figure 2:**
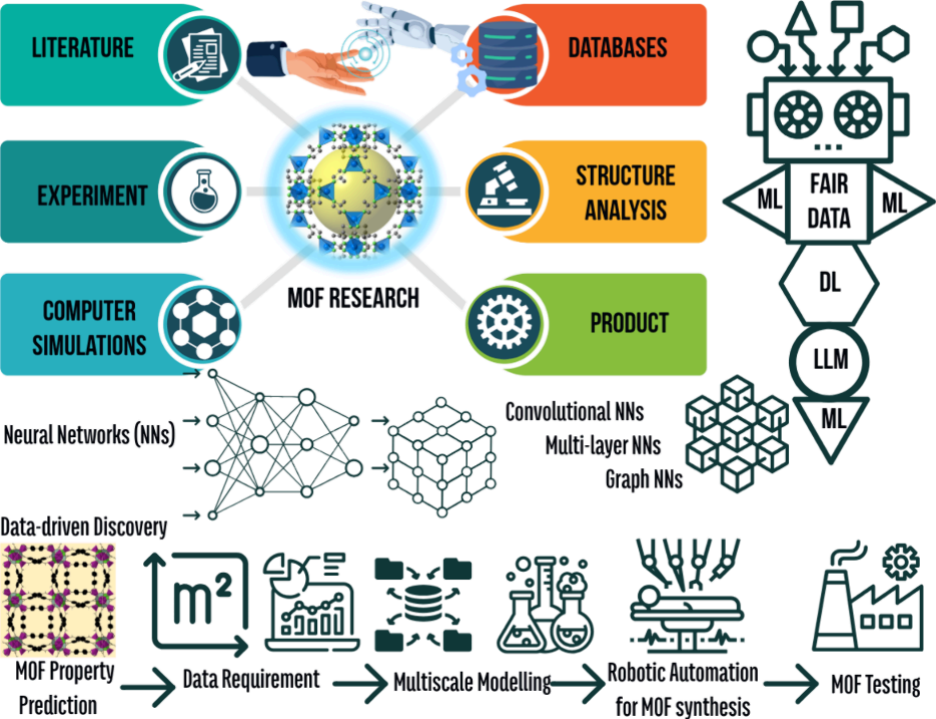
Interplay within the overall AI paradigm for the progress of MOF research.

Data-driven approaches reduce the need to run molecular simulations for every material, and integrating ML into molecular simulations and experiments has significantly accelerated the MOF discovery process in the last years. However, most ML and molecular simulation studies have primarily focused on gas adsorption under moderate to high-pressure conditions. A limitation remains the lack of accuracy in these approaches for more complex energy-related applications, particularly those involving gas capture at low traces, such as direct air capture (DAC) or adsorption of highly volatile compounds. By applying innovative derivative-free optimization methods such as Bayesian optimization^42^ and multifidelity methods,^43^ and sophisticated techniques such as new neural network (NN) architectures, ML can analyze vast MOF databases to identify key structural patterns associated with desirable properties, such as high gas adsorption, selectivity, or thermal stability. For example, Liu et al.^44^ determined the ML hyperparameters via Bayesian optimization and used a crystal graph convolutional NN algorithm to virtually screen MOFs for toluene vapor adsorption. This narrows down the candidates for experimental synthesis, guiding researchers directly to promising compounds and expediting the discovery process. Several reviews^45–51^ reflecting aspects of data acquisition, featurization, ML model training, and applications have already been published.

Deep learning (DL),^52^ a subfield of ML based on NNs, has revolutionized the AI field with its impressive results in applications such as computer vision, natural language processing and speech recognition. One of the most important factors for the success of DL algorithms is the availability of large data sets like ImageNet,^53^ since these algorithms are notorious for being “data hungry”. With this in mind and taking into account the development of large MOF databases,^54–61^ the appearance of DL techniques within MOF research should not be a surprise. DL algorithms enable researchers to directly process text-, graph- and image-based representations of MOFs or even raw structural information.^62–64^ A variety of DL workflows, based on multilayer perceptrons,^31,65–70^ recurrent NNs (RNNs),^68,71–73^ graph NNs (GNNs),^57,74–80^ convolutional NNs (CNNs)^62,81,82^ and transformer-based NNs,^83–86^ have been developed and successfully applied for predicting various properties of MOFs: gas uptake,^62,83,84,86,87^ gas diffusivity,^84^ band gap,^83,84^ bulk modulus,^65^ stability metrics^70^ and synthesizability.^71^

Besides uncovering structure–property relationships, predictive DL models can also be applied for accelerating expensive steps in computational workflows.^74,79,87–90^ For example, modeling nonbonded interactions in Monte Carlo or molecular dynamics simulations of gas adsorption/diffusion requires accurate partial atomic charges for all MOF atoms. For example, Raza et al.^74^ proposed a GNN that takes as input a crystal graph—i.e., a set of nodes and edges, representing atoms and bonds between atoms, respectively—and which generates node-level predictions, corresponding to partial charge predictions for MOF atoms while satisfying the charge neutrality constraint. The GNN was trained with DFT (density functional theory)-derived MOF partial point charges, achieved high-fidelity partial charge assignment, and importantly, with orders of magnitude shorter runtime compared to DFT calculations.

Other than predictive models which have been widely applied for high-throughput screening, generative models such as Generative Adversarial Networks (GANs) and Variational Autoencoders (VAEs) are other classes of ML models that can expedite the discovery of high-performing MOFs. Instead of mapping from structure-to-property (as is the case of predictive models), these models adopt an inverse design^73,80,91^ approach (i.e., property-to-structure), enabling the targeted design of tailor-made materials.

The long-term vision is to combine these data-driven predictions with real-time feedback loops and autonomous laboratory systems. In the experimental space, DL could integrate with robotic automation for MOF synthesis, enabling fully autonomous laboratories where DL models not only design new MOFs but also control robotic systems to synthesize and test them.^92,93^ This would drastically accelerate the pace of discovery. Additionally, robotic and automated chemistry laboratories can produce vast amounts of data, underscoring the need for effective learning methods to process them. At this point, we note a key dilemma in MOF research: the difficulty of automating experimental processes. Many synthesis protocols lack reproducibility, often yielding inconsistent results even when the same procedure is followed. In addition, each research group employs specialized experimental procedures, making integration into a commercially available automated tool challenging. These compatibility issues are not only coming from materials synthesis but also from available software infrastructures. For example, in a *Nature Synthesi*s Q&A discussion,^94^ Prof. Andrew Cooper highlighted that key barrier to automating material synthesis is not the cost but rather specialized expertise required to implement generalized experimental systems. For example, his laboratory uses the Robot Operation System (ROS), yet compatibility issues persist, as not all robotic platforms are ROS-compatible. Additionally, challenges remain in standardizing software libraries and their interface programming, further complicating widespread adoption. The flexible automation concept^95^ may solve these experimental challenges by dividing the workflows into individual tasks such as synthesis, activation, stability testing, and measurement. This task-oriented approach can introduce reconfigurable automated dynamic experiments.

From the materials synthesis perspective, reinforcement learning (RL) adds another layer of precision by dynamically optimizing synthesis parameters—including temperature, solvent choice, and reaction time—to achieve higher yields, enhanced crystallinity, and better phase purity. For example, Yaghi’s team^26^ developed an integrated AI system to determine the optimal conditions for the synthesis of MOFs and their organic related materials, covalent organic frameworks (COFs), for water harvesting in his laboratory. Microwave-assisted methods required 4 days (6,235 min) to optimize one compound from over 6 million variable combinations. This adaptive optimization could minimize trial and error, especially in challenging syntheses, and can unveil synthesis conditions that may otherwise remain unexplored. In another example, a web-based tool was developed to predict MOF synthesis conditions using ML models.^96^ Users can upload the crystallographic files of MOFs and then receive the synthesis conditions of the corresponding MOFs, including synthesis temperature, time, solvent, and additives. These studies introduce a transformative approach in MOF synthesis, moving from experience-driven trial and error toward a systematic inverse design strategy.

The integration of AI tools significantly reduces operational costs by minimizing labor hours, reagent consumption, and equipment usage. To illustrate this, consider the optimization of UiO-66 synthesis. In 2020, Taddei et al.^97^ performed 31 experiments to optimize the microwave synthesis conditions, achieving a significant increase in space-time yield (STY) from 23 kg/m^3^·day to 2241 kg/m^3^·day. The production cost of 1 kg of activated UiO-66, synthesized using dimethylformamide, zirconium chloride, terephthalic acid, and hydrochloric acid, has been reported as approximately 503.9 USD/kg.^98^ For 31 reactions, each yielding 360 mg of UiO-66, the total product mass is 11.16 g, resulting in a material cost of 5.62 USD per batch. The power consumption for these 31 experiments was reported as 2438 W. Assuming a total experimental duration of 30 days and an electricity cost of 0.15 USD/kWh, the energy cost totals 263.3 USD. If the experimental work is conducted by 5 PhD students (each with an estimated salary of 2500 USD/month, totaling 2.5 full-time equivalents), the labor cost totals 6250 USD/month. Combining these factors, the total estimated cost for this workflow is 6518.92 USD/month. To reduce costs, AI-driven optimization was explored. By employing AI tools (assumed cost: 2000 USD/month such as subscription fees, computational resources, maintenance, data storage, power consumption etc.) and requiring only 1 PhD student (0.5 full-time equivalent) to perform an optimized synthesis (producing 360 mg per experiment), the total cost is reduced to 3250.18 USD/month (including labor, materials, and AI implementation). This represents an almost 50% reduction in overall cost compared to the conventional approach. Furthermore, multiscale modeling—combining DL models that operate at different scales (from atomic to macroscopic)—could enable researchers to predict how MOFs would behave in real-world conditions, aiding in their deployment in industrial applications.^99^ The potential impact is vast, with applications in CO_2_ capture, hydrogen storage, water purification, and beyond—ultimately enabling a new era of responsive, high-performance materials tailored to tackle some of the world’s most pressing challenges.^100^

Figure 2 highlights the growing concerns about AI potentially replacing human-centric jobs. While such concerns are valid, it is important to recognize that disruptive technologies have consistently brought both challenges and opportunities. By focusing on collaboration and innovation, scientists and society overall can harness the potential of AI for great achievements. Ultimately, as data-sharing platforms expand and interdisciplinary collaborations grow, the synergy between AI and MOF research could revolutionize materials science, enabling breakthroughs in clean energy storage, environmental remediation, and beyond. The future of AI in MOF research is not just about better predictions but about unlocking the ability to design materials with tailored properties on demand. Additionally, future research should not only focus on beating state-of-the-art results but should also provide practical and environmentally sustainable AI solutions. Thus, new researchers are encouraged to report the computational and carbon footprint^101,102^ of their proposed approach in addition to standard performance metrics. We believe that AI is a pivotal tool in accelerating the journey toward novel MOFs and the recent breakthroughs are just the tip of the iceberg.

## From Early AI Predictions to the Era of Large Language Models

Large Language Models (LLMs) are potential game changers within the rapidly expanding research space of AI. The methodology of LLMs involves training NNs on vast amounts of text data, enabling them to understand, generate, and reason about human language. Figure 3 shows human-interpretable LLMs for MOF research.

**Figure 3:**
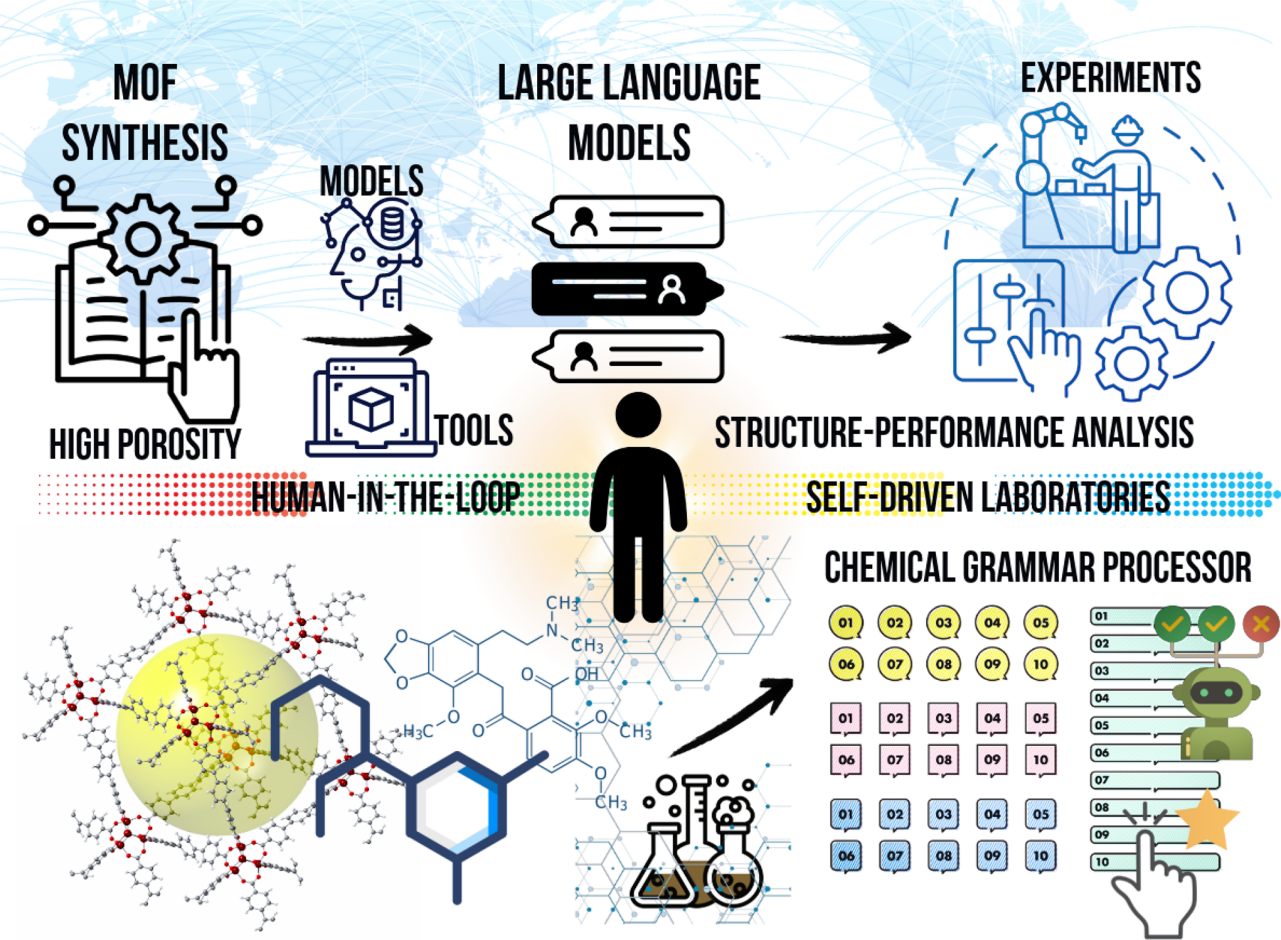
Possible AI-human loop for MOF chemistry.

LLMs can be used in MOF research in several innovative ways: (a) They can automate literature reviews by scanning large data sets and extracting key insights from research papers, making it easier for researchers to stay updated on the latest developments. (b) LLMs can generate structured data from textual descriptions, aiding in the discovery of new MOF structures or optimizing synthesis methods. (c) Through integration with ML models, LLMs can predict experimental outcomes, propose new materials, and link disparate sources of information, leading to interdisciplinary breakthroughs. (d) Additionally, they can optimize experimental conditions by analyzing previous experiments and suggesting new approaches, accelerating the discovery of MOFs with desirable properties. Independent of these applications, LLMs hold potential for research automation, such as drafting reports, summarizing results, or hypothesizing new experiments based on available data. Fine-tuned on MOF-specific data and integrated with existing databases, LLMs can enhance information retrieval and facilitate collaboration across different fields, ultimately driving innovation and accelerating progress in MOF research.

To bring them to a wider audience and use them in service of more people, we need to identify the kind of tasks where LLMs are superior: LLMs are particularly good at summarization, sentiment analysis and text classification. For example, LLMs can analyze vast databases of scientific publications to identify trends and extract critical information on material properties, synthesis methods, and experimental results. This capability allows researchers to quickly gather comprehensive insights without manually skimming through thousands of papers. This would be the most obvious short-term adaptation of LLMs directly to MOF research. Implementation with a wider impact and a longer-term view would suggest the need to create tools and methodologies to convert materials synthesis and chemistry into a “language” and to teach the LLMs the “grammar of chemistry”. The massive ML research effort of recent years has already created a great portion of the necessary tools, such as molecular representations, reaction descriptors, retrosynthetic analysis, condition optimization and a significant amount of data output.^23^ So, what would be the steps of creating and converting an LLM for MOF topology generation and synthesis as a “reasoning engine”?

There are emerging answers in the literature converging on this fundamental question. One example is called ChatMOF, created by Kang and Kim.^7^ ChatMOF can create MOFs with user-desired properties from human cognition and predict their properties. For example, ChatMOF can not only answer a text-compatible input, but also generate a MOF structure with user-defined properties. ChatMOF extracts the desired MOF data using a table-search operation from different MOF databases such as CoREMOF,^56^ the CSD MOF subset^103^ or QMOF^57^ and also uses MOFkey^104^ and DigiMOF^58^ databases to provide topology-based and synthesis information. MOFTransformer^84^ is used as a toolkit to predict the properties of MOFs based on an ML model. Here, it is important to clarify that ChatMOF leverages language to utilize knowledge already curated in its data set, which differs from actual reasoning on a chemical task or question—a capability that remains a challenge for AI.

To dive into the discussion for adapting a LLM for MOF topology generation, the first step is to create a way of generating chemical word embedding and tokenization. Chemical word embedding is a database structure which stores chemical words (ligands and metals in terms of MOFs) according to some proximity rules and locates similar words nearby and dissimilar words far apart. What makes two ligands similar in terms of reactivity, MOF synthesis or output topology, i.e., in their “meaning”? That seems like an open question waiting for a rigorous answer.

This issue was sorted out in Natural Language Processing (NLP) research by word2vec,^105^ an analogous methodology and development for MOFs (maybe named: MOF2vec) seem desirable. Additionally, a relevant methodology for tokenization is needed for a MOF reasoning engine. In NLP, tokenizing a given text word-wise overloads the vocabulary dictionary (a sequence assigned to each word). On the other hand, tokenizing a given text character-wise results in a very lightweight vocabulary dictionary (a number assigned to each letter and the list has only 26 items in English). But this time, the context within words is lost. Therefore, in LLM applications, tokenization is a well-tuned process. The question we need to answer is how to fragment a given MOF structure to generate a “sub-word” dictionary that would enable us to speak the language of MOF chemistry.

Word embeddings of chemical elements are used to represent the stoichiometric formula of MOFs, and the chosen embeddings are derived from unsupervised learning on raw text (i.e., natural language texts) to capture implicit knowledge from the corpus (a large and structured collection of texts in a natural language). To construct features based on the composition of each MOF structure (almost 200 embedding dimensions), the ElementProperty featurizer in matminer^106^ is utilized. This model facilitates material design by establishing a connection between property predictions and crystal structure to further develop high-performance materials for gas adsorption applications. Geometric entities can be used to predict some chemical properties of MOFs, but given the large variety of MOFs, how can one create a chemistry-based design using a MOF language? We already have some tools and the vision to harness LLMs as a “chemical grammar processor”. Although the road to this goal might not be direct, each new tool brings us closer to the rational design of new materials.

The future perspective of LLMs in MOF research holds immense potential to revolutionize how we design, discover, understand, and, more importantly, think about these materials. As LLMs become more sophisticated and fine-tuned for specialized scientific domains, they will likely evolve into essential tools for automating much of the research process. In the coming years, we could see LLMs used for real-time hypothesis generation, where researchers can interact with the model to brainstorm new ideas, identify unexplored research avenues, or propose novel MOF structures with specific properties, such as enhanced gas adsorption or catalytic efficiency. This will significantly shorten the discovery-to-deployment cycle for new materials.

Moreover, as LLMs become better at integrating vast and diverse data sets, including experimental data, computational models, and scientific literature, they could act as highly intelligent systems capable of predicting material behaviors under various conditions. These models may go beyond summarizing existing research to synthesize new knowledge by connecting dots across different scientific domains. This interdisciplinary approach will likely unlock new applications for MOFs in areas like renewable energy, environmental remediation, and drug delivery, as LLMs provide insights that human researchers might overlook.

Looking further ahead, LLMs combined with other AI technologies could enable fully autonomous research pipelines. In these systems, LLMs would not only generate hypotheses but also design and execute simulations, analyze results, and even control robotic systems to perform physical experiments. This could lead to the discovery of completely new classes of materials with tailored properties, optimized for specific industrial or environmental applications. Since industrially relevant conditions are critical for assessing the environmental impact of MOF products,^107^ AI can help explore the infinite number of scenarios such as varying reaction conditions, solvent recovery or waste management processes. Ultimately, LLMs could transform MOF research by making it faster, more efficient, and more innovative, pushing the boundaries of what is currently achievable in materials science.

In the past years, the many potential applications and varieties of MOFs have truly come to the fore. One reason for this blossoming of the field is the development of LLMs and generative AI tools that can support scientists in searching the reaction space for this class of materials. What these efforts make clear is that digital standardization of terminology, structural representation, and nomenclature is essential for harnessing the full capabilities of digital tools. Having a standardized nomenclature in the MOF community is important for ensuring interoperability between data management and software systems. These backward-compatible standards are only the first step in creating a common language. As the chemistry community, we must also ensure that there is universal dissemination and uptake. This will enable us all to speak the language of MOFs and the language of chemistry!

Learning algorithms have been proposing solutions not only for data collection, interpretation, and structuring, but also for a long-standing problem of the molecular modeling community called “scale hierarchy”. Scale hierarchy problems refer to the difficulty of integrating results from the electronic-level simulation to the atomistic level, from the atomistic level to the mesoscale, and from the mesoscale to the continuum level. To overcome this difficulty and plug the gap between the “electronic level to the atomic level”, physical models were commonly used and had limitations. Now, ML-based potentials open up completely new possibilities to tackle this problem.

## ML Potentials: A New Frontier in Atomic-Scale Simulations of MOFs

Interatomic potentials are critical in understanding atomic interactions and predicting the properties of materials at the atomic scale. Traditional quantum mechanical-based methods such as *ab initio* methodologies have delivered essential insights, however they are limited in terms of time and length scales. Alternatively, empirical or semiempirical models, such as Lennard-Jones or embedded atom potentials, have been extensively used in the past two decades to describe the intra- and intermolecular interactions in MOFs. However, these classical potentials often struggle to balance accuracy, computational burden, and generalizability across diverse chemical MOF systems. Machine learning potentials (MLPs) represent a new avenue for capturing the most complex interactions with near quantum mechanical-accuracy but at a fraction of computational cost. This has enabled studying MOF materials with large atomic-scale simulations that were unfeasible at the quantum mechanical level, from exploring phase transitions to predicting their mechanical, thermal and adsorption properties among others. Unlike classical potentials, MLPs are trained on high-quality quantum mechanical data, often derived from DFT or other advanced *ab initio* methods, assembling a large data set of atomic configurations and associated energies/forces of the explored MOF systems.^108,109^ As illustrated in Figure 4, by employing ML algorithms ranging from NNs to Gaussian regression or kernel methods to map atomic positions to the potential energy surface, these MLP models can capture the intricate atomic interactions, including bond breaking and formation, with unprecedented precision. By continuously learning from larger data sets and adapting to various atomic environments, MLPs show a high adaptability because they can be improved by incorporating new data. This makes them highly versatile for simulating MOFs with different compositions, structures, and environmental conditions. MLPs can be easily applied to more complex systems, something that has been a limitation of traditional models. Thus, disordered or amorphous phases can be modeled, providing unprecedented insight into the flexible and dynamic nature of materials.^110,111^

**Figure 4:**
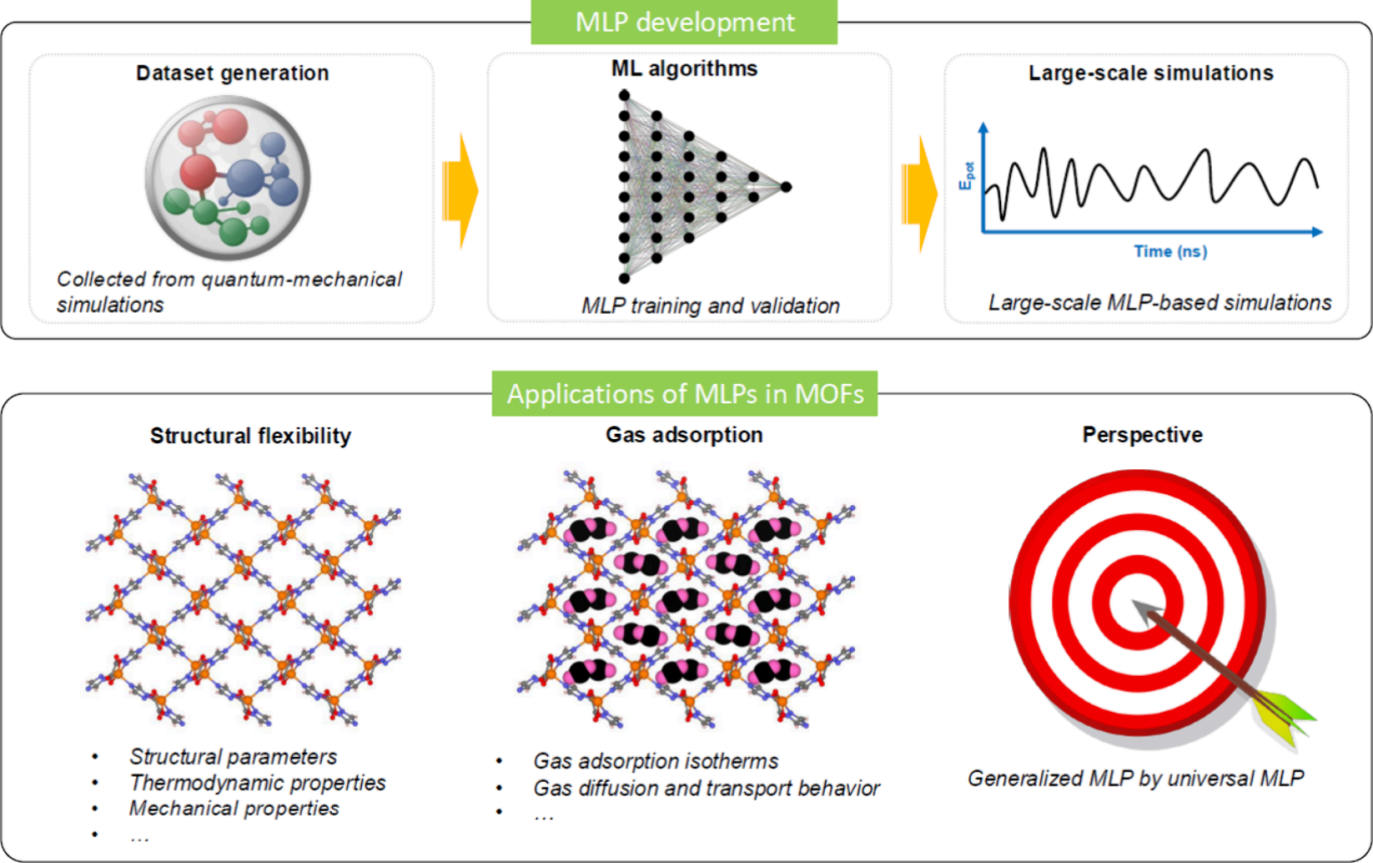
Schematic diagram of the development of MLPs based on quantum mechanical data sets alongside typical applications of MLPs to the MOF field.

In recent years, the use of MLPs in the field of MOFs has witnessed a profound and rapid expansion, improving the exploration of the physical properties of this class of materials by unlocking deeper insights. Decisively, a precise description of the structural flexibility of MOFs via MLPs offers a unique opportunity to explore how these hybrid frameworks respond to varying temperature and pressure. Some typical illustrations include the exploration of the zeolitic imidazolate frameworks (ZIFs),^112^ MOF-5,^113^ CALF-20,^114^ and 2D MOFs.^115^ For example, MLP-based molecular dynamics simulations revealed unique thermodynamic and mechanical properties of CALF-20 at finite temperature, e.g., negative area compressibility, negative thermal expansion and unusual strain-softening behaviors.^7^ Another advantage of MLPs is the ability to simulate large systems at experimentally relevant scales. Unlike DFT calculations, which are limited to small systems and short time scales, a recent study demonstrated that a high-quality MLP trained for a 2D MOF can be used to simulate experimental-size MOF membranes (up to 28.2 × 28.2 nm^2^) without losing computational accuracy.^115^ Thus, the behavior of MOFs can be studied under conditions closer to practical applications.

MLPs have also been applied recently to predict the gas adsorption properties of MOFs, e.g. Al-*soc*-MOF-**1d** and ZIF-8.^87,116^ with high precision. Here, one of the most critical challenges is to accurately describe the host/guest interactions. In classical simulations, the van der Waals interactions, typically modeled by Lennard-Jones and Buckingham potentials with parameters taken from generic force fields, e.g. UFF^117^ and DREIDING,^118^ are augmented by an electrostatic term to account for the interactions between charged MOF atoms. However, there are many examples where force fields must be reparameterized or derived from quantum-mechanical calculations due to a poor agreement with experiments, especially when the MOF framework contains open metal sites (OMSs).^119,120^ Decisively, MLPs can accurately model not only the most complex MOF/guest interactions but also the guest-induced dynamics of the MOF framework which is most often overlooked in classical simulations with the use of rigid-lattice models. Typically, a MLP trained on a relatively large data set of MOF/guest configurations generated by *ab initio* molecular dynamics was demonstrated to accurately capture the H_2_/Al-*soc*-MOF-**1d** interactions as well as the H_2_-triggered MOF scaffold dynamics leading to a predicted adsorption isotherm at 77 K via grand Canonical Monte Carlo simulations in excellent agreement with the experimental data.^87^ This strategy could be generalized to provide a more accurate and efficient assessment of the adsorption behavior of the most complex MOFs.

Typically, an MLP is mostly trained on a single MOF phase and therefore struggles with transferability across different chemical and structural environments. This would lead to inaccurate predictions for other MOFs. The breakthrough in this field would therefore be the development of universal MLPs to model the vast structural diversity of MOFs with quantum mechanical-level accuracy. The integration of such highly accurate MLPs with HTCS tools would enable researchers to anticipate a myriad of MOF properties in real-time and with unprecedented precision. In this context, the ongoing development of universal MLPs, e.g., CHGNet,^121^ M3GNet,^122^ MACE,^123^ holds immense promise. Another key evolution lies in the development of self-improving MLPs, where models continuously learn and adapt from new data, expanding their predictive power across an ever-wider range of MOFs. This could drastically accelerate the discovery of novel MOFs for next-generation applications by exploring vast MOF chemical/structure spaces.

To be able to use all these fascinating methods, we initially need high-quality data. This is a tedious problem since the collection and precollection are still not very well-defined to reach “high-quality” data. That is why we would like to insist again that the major problem here boils down to, inspired by Sherlock Holmes, the famous fictional detective created by Sir Arthur Conan Doyle:^124^ *Data, data, data! I cannot make bricks without clay.* In this context, we would like to identify some key entry barriers for data-centric design as well as suggest some systematic precollection and collection methods to reach high-quality data for MOF research.

## Lowering the Barrier to Entry for Data-Based Materials Design

As highlighted above, we are witnessing a rapid explosion of the number of proposed scientific methods based on data for the discovery of novel materials, the identification of known materials with specific desirable properties, the optimization of chemical engineering processes and, more broadly speaking, multiobjective optimization and decision making in the field of applied materials sciences. Accompanying this fast pace of theoretical development, there is an important demand from the wider research community — and not only experts in data science — to be able to use these methods that they hear so much about (both in scientific publications and in the general press), with legitimate questions such as *“If AI and/or ML is going to change the way of chemistry, how can I leverage it in my own research projects?”*. We see in the field a growing recognition of the need to democratize access to data — and beyond data, to models.

The drive to lower the barrier to entry for the use of data-based methods is 3-fold, in our view: (i) it concerns the access to data, (ii) the access to models, (iii) and their ease of use to the wider community. All three aspects are necessary to drive the adoption of data-based methods in the materials science research community, beyond data scientists and specialists in theoretical and numerical methods.

The first aspect that we highlight is the sharing of data, to maximize the reuse of research data. This need is driven not only by research ethos but also by an increased recognition that there is a clear economic cost associated with dark data or unshared data – which was costed at €10.2bn per year at the scale of the EU economy.^125^ We note again that probably the best-known set of guiding principles for sharing scientific data in the modern age are the FAIR principles.^126^ The FAIR data standards are Findability, Accessibility, Interoperability, and Reusability. While many research groups nowadays make efforts to make their data findable and accessible, we emphasize that interoperability and reusability are sometimes more difficult to achieve, especially in a field where the nature of the data (and the materials that are described) are incredibly diverse. However, interoperability and reusability are of absolutely fundamental importance. Progress in this area will require work to improve the metadata associated with the data itself, their representation and standardization, as well as the use of shared, accessible vocabularies and ontologies. This touches on the core issue of “What defines a material?”, a question that different research communities (and families or classes of materials) would have different answers to.

The second aspect necessary to democratize data-based methods is that of access to models, i.e., to the trained ML models, to the code that was used to train them, and to the code that is necessary to deploy them. This is crucially important for the reproducibility of scientific research in our field, and a cornerstone of the scientific method. Without full sharing of models, it is impossible (for groups other than the original authors) to benchmark published methods on new data, or to compare the merits of different algorithms — and therefore an obstacle on the road to progress. This aspect is also of vital importance to bolstering accountability in AI research, something that is necessary to build the trust of the broader community. Furthermore, it is also linked, to some level, to a necessary homogenization of the reporting standards for new data-based studies in our field. We hope that in the future the community in materials sciences and chemistry will propose and enact coherent reporting requirements. We note that such efforts have been proposed before, either in the form of best practices by experts in the field,^127^ or through funder mandates (similar to open access mandates).^128^

The third prong of this push toward democratization of data-based methods may appear, on first view, as less “scientific” or technological than the previous two. However, we argue that there is an important demand to make published data and models easier to use for the wider community, through initiatives like centralized databases with online portals, user-friendly data visualization tools, training programs, and data analytics programs. For example, while many MOF databases have been proposed, they are often hosted on different platforms (some on Zenodo, some on GitHub, some on specific websites, etc.). One example of the push toward unification (and therefore, greater interoperability) is that of the Materials Project web portal.^129^ The Materials Project portal is an open web-based resource of computed properties of materials. It is centralized but it also allows for the upload of user-created data in the form of external “contributions”, allowing better sharing of data through a common platform with a well-defined user interface. Future efforts should be encouraged for such platforms, extending to a wider range of chemical space (beyond crystalline materials, for example) and suitably integrating both experimental and computational data of both physical and chemical nature, further enabling interdisciplinary collaborations. Similarly, published models can be integrated into such online platforms, making it easy for nonexperts to perform simple property prediction (or other ML tasks) simply by uploading one or several structure files. This would allow large-scale screening of both experimental and hypothetical structures to identify promising candidates by pushing the Pareto front for specific applications, in a multiobjective optimization strategy — possibly, in the long term, including questions that are deemed too difficult at the moment, such as generative models for materials design and realistic estimation of synthesizability (or feasibility) of hypothetical structures.

This whole task is an aspirational but necessary attempt to democratize the landscape of data and AI methods for the new generation materials development endeavor. How can we adapt all these suggestions and guiding principles into a vibrant ecosystem of MOFs, COFs, and similar materials?

## Creating an Integrated Material Database

Responding to this challenge, we suggest to integrate existing material databases into an overarching, curated platform and diversifying its coverage through three distinct steps. This suggestion is based on recent evaluations of existing databases—both experimental and computational in nature—that uncovered important shortcomings, as discussed by Gibaldi et al.^130^ and De Vos et al.,^131^ as well as on personal experiences of developing and using these databases.

### Step 1. Data Collection and Integration

In the past decade, a wide variety of MOF and COF databases based on experimental structures,^56–58,56–58,132–134^ hypothetical structures,^54,55,131,135–137^ or a combination thereof^59^ have been developed, often as a starting point for high-throughput screening studies. These MOF and COF structures, once properly represented using, e.g., the MOFid format^104^ or the Weisfeiler-Lehman kernel in the graph2vec algorithm,^138^ form a promising starting point to establish the integrated database platform envisioned here. In this platform, the tokenized reticular structures, properties reported in the original database, and, additionally, properties such as pore size distribution,^139^ persistence diagrams,^140^ and revised autocorrelation (RAC) descriptors^141^ that are calculated separately from the original database, would form individual ‘documents’, as in the MOF recommendation system established by Zhang et al.^142^ Such standardized document-structured genomes^140^ ensure that the database can afterward be leveraged to recommend candidate materials for specific applications based on the similarity of their material embedding vectors with embedding vectors of known well-performing materials generated through a Doc2Vec model. In addition, once the materials are tokenized, this database can be hugely expanded and enriched through text-mining the existing MOF and COF literature, similar to the DigiMOF database.^58^ This algorithm could cover new literature continuously, ensuring the platform remains up-to-date.^58^ For both the original data points and those obtained by text mining, sufficient metadata must be present, including the source of the data, whether it was experimentally verified, computationally calculated, or predicted by a model, and the context in which the property was reported.^141^ This would help adhere to the FAIR principles and identify and correct the “fuzzy” context omnipresent in materials science.^142^

### Step 2. Data Correction and Curation

At this point, duplicate structures may be present in our database, which would bias subsequent model training.^143^ The database would likely contain errors, either because they were present in the literature, or because they were introduced during text mining. Recently, MOSAEC-DB (Metal Oxidation State Automated Error Checker Database),^130^ a database containing over 124,000 MOF structures, was shared within the MOF community to address structural problems arising from computational processing, such as those caused by solvent removal or unreasonable assigned oxidation states. These contributions are highly appreciated, as they establish a unified platform to support MOF research. While it is straightforward to remove (near-)­duplicates based on their overlapping embedding vectors, correcting and enriching data requires more attention. A possible path forward here is based on the observation that the collective knowledge in large databases can help correct mistakes in individual entries, as demonstrated by Jablonka et al.^144^ To do so, various competing ML models would be trained and tested on our database’s existing {material, property} pairs and then used to predict these properties for the whole database. In most cases, this is new information that enriches the database, while a limited amount of material predictions can be compared with actual experimental or simulation outcomes. This step is vital to test the accuracy of the ML models and to help identify possible errors in the database, as in ref.^144^, especially when the predictions of multiple ML models agree with one another. In cases where these models disagree, domain experts would still need to identify the most likely result.^25^

### Step 3. Data Diversification

Most hypothetical databases are biased due to the limited amount of building blocks and topologies to generate hypothetical structures, while both literature studies and experimental databases tend to be skewed toward easy-to-synthesize materials.^131,141^ To ensure our platform is sufficiently diverse and can be adopted to identify promising materials beyond the limited chemical space explored until now,^143,145^ it is essential to assess its variety, balance, and disparity in terms of the properties calculated in Step 1.^141^ By identifying unexplored and weakly explored regions in chemical space, hypothetical MOF and COF structures could be generated through, e.g., LLM-based genetic algorithms targeting these regions, as demonstrated recently.^7,23,146,147^ Besides the excellent integration between LLMs and genetic algorithms for this inverse design task,^7,23,146,147^ this also allows for the efficient exploration of the structure space of crystalline materials by varying the temperature in the final softmax layer.^23,148^ Such hypothetical structures can be straightforwardly generated using the reticular principle by extracting and recombining building blocks, as demonstrated before,^145,147^ paying specific attention to defective and disordered structures, given their profound impact on the resulting material properties.

## Conclusions

The 2024 Nobel Prizes in Physics and Chemistry both celebrated groundbreaking advancements in AI, physics, chemistry, and computational methods that deepen our understanding of complex structures. If we turn to MOF chemistry, the integration of ML, DL, and LLM has shown remarkable potential in advancing material research. By accelerating the discovery process, predicting material properties, and enabling efficient data analysis, these tools address complex challenges in MOF design and optimization. The use of ML and DL algorithms can significantly reduce experimental time and resource costs, while LLMs support researchers by quickly summarizing relevant literature, hypothesizing new MOF structures, and even generating new pathways for synthesis. Together, these approaches are making MOF research more vibrant, insightful and data-driven.

Looking ahead, the application of ML, DL, and LLMs in MOF research is expected to expand significantly. Figure 5 summarizes the extraordinary impetus of AI for new materials development in the field of porous materials, specifically MOFs. Future efforts will likely focus on creating more specialized models tailored for complex MOF systems, integrating multimodal data for enhanced predictive capabilities, and improving model interpretability to deepen our understanding of MOF behavior at the atomic and molecular levels. As these tools become increasingly accessible and sophisticated, they may eventually support real-time, AI-driven experimental design, fostering an era where MOF discovery and application reach unprecedented heights. This synergy between AI and materials science promises to catalyze transformative advancements across fields such as energy storage, catalysis, and environmental remediation by using MOFs.

**Figure 5:**
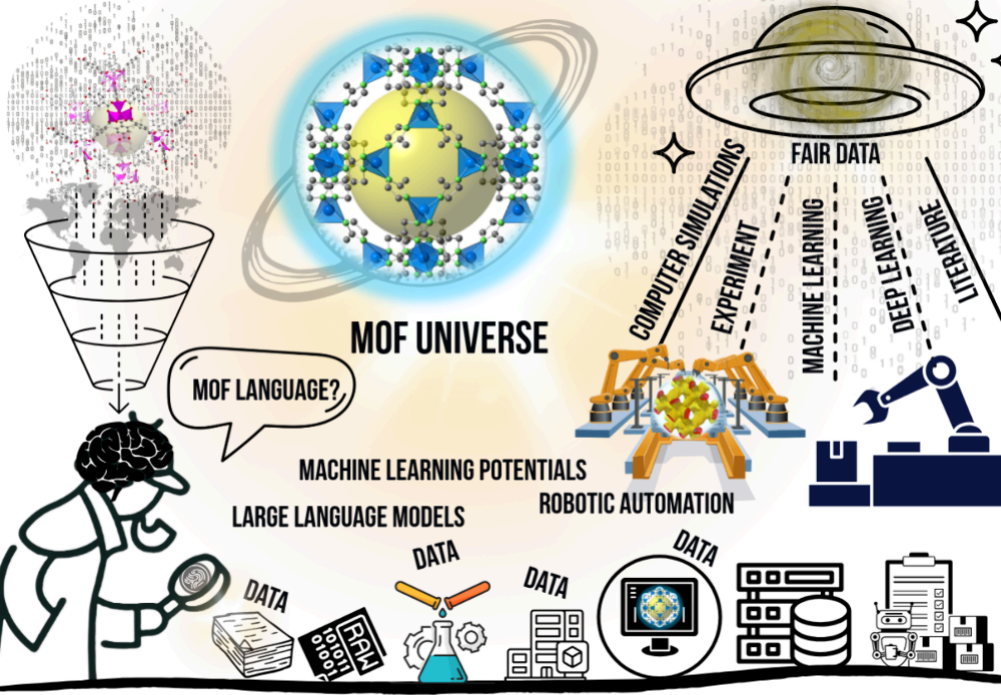
Future progress of MOF research.

There is still much room for exploring the vast chemical spaces of materials and their flexible/stable configurations. Predictive modeling remains in its infancy for applications like healthcare and energy storage. Integrating AI with automated MOF synthesis for scale-up technologies, especially using green chemistry principles can enable tailored solutions, such as high-performance batteries or personalized cancer treatments. These are only a few examples, and the possibilities are endless.

Overall, with the increasing importance of data-driven decision making and the proliferation of (AI-driven) chatbots, there is a growing recognition of the need to democratize access to data. The MOF community should find ways to remove the barrier to data access and use by providing user-friendly databases, data visualization tools, training programs, and data analytics programs. Cross-functional collaboration should be encouraged by supporting data-driven initiatives. These provide vital goalposts to be reached in the area of material design with AI.

We believe that with the potential of human creativity and the power of experimental design and modeling tools, the sky is the limit!

## 12

Springer Nature
